# Psychological Experiences and Motivations of Oocyte Donors in Spain

**DOI:** 10.5935/1518-0557.20260051

**Published:** 2026

**Authors:** Marta Guerri Pons, Natalia Romera Agulló

**Affiliations:** 1 Department of Psychology, Clínica Fertty, Barcelona, Spain; 2 Department of Psychology, Instituto Bernabéu, Alicante, Spain

**Keywords:** oocyte donation, donor motivations, psychological well-being, assisted reproduction, anonymity

## Abstract

**Objective:**

To analyze the psychological and emotional experiences of women who donated oocytes in Spain.

**Methods:**

A cross-sectional observational study was conducted between June 2024 and February 2025 at Instituto Bernabéu (Alicante) and Clínica Fertty (Barcelona). The sample consisted of women who met the inclusion criteria for oocyte donation programs. Participation was voluntary and anonymous, with informed consent obtained. The study was approved by the internal research committee of Instituto Bernabéu and registered under protocol MR48, in accordance with the Declaration of Helsinki. Data were collected using the EMODON questionnaire, developed ad hoc for this study, which assessed motivations, concerns, perceived professional support, and emotional state. Quantitative data were analyzed descriptively, and qualitative responses underwent thematic analysis.

**Results:**

The mean age was 25 years (SD=3.88). Most donors reported altruistic motivations (95%), while 61% cited financial compensation. Concerns before treatment were expressed by 55%, primarily regarding future fertility (70%), hormonal side effects (50%), and surgical risks (45%). During stimulation, 54% felt emotionally stable, 24% more irritable, and 13% reported a negative emotional impact overall. After donation, 92% felt calm and satisfied. Foreign donors reported higher irritability (34%) than Spanish donors (19%). Nearly all participants (99%) valued the professional support received. Regarding anonymity, 71.3% stated it would not affect them or had never considered it, while a minority reported concern or refusal to donate again.

**Conclusions:**

Oocyte donation was generally well tolerated emotionally, with high satisfaction regarding clinical support. Nonetheless, a vulnerable subgroup highlighted the need for targeted psychological assistance.

## INTRODUCTION

Unlike other forms of donation, such as blood or bone marrow, gamete donation-and oocyte donation in particular-has remained relatively invisible and socially surrounded by stigma and limited awareness. Information about these programs often circulates through informal networks, and many women preferred to keep their participation secret, even from close family and friends. In clinical practice, donors were usually approached from a biomedical perspective, focusing primarily on their role as providers of genetic material, without receiving emotional support equivalent to that offered to recipients.

In recent years, the number of assisted reproduction treatments in Spain has steadily increased, driven by delayed motherhood and the diversification of family models. According to DBK Informa (2024), the number of fertility centers in Spain has grown significantly, consolidating the country as a benchmark in this field. Data from the National Registry of the Spanish Fertility Society (SEF) indicate that in 2022 a total of 39,546 babies were born through assisted reproductive techniques, representing approximately 12% of all births in Spain. Of these, 7,882 were the result of oocyte donation, accounting for about 22% of all live births achieved through assisted reproduction (SEF, 2024).

Donors are young women who voluntarily undergo a medical procedure motivated by a combination of factors: the desire to help others, financial compensation, or even the opportunity to learn about their own fertility (Hudson *et al*., 2019; Lima *et al*., 2020). Although their participation is essential for the success of fertility treatments, the emotional and psychological experiences of oocyte donors during the process have received limited attention in the scientific literature.

This study aimed to analyze and comprehensively understand the experiences, motivations, and concerns of oocyte donors in Spain, with a particular focus on the psychological and emotional dimensions that arose during the donation process.

## MATERIALS AND METHODS

We designed a cross-sectional observational study with the objective of describing and analyzing the psychological and emotional experiences of women who participated in an oocyte donation program in Spain. Data collection was conducted between June 2024 and February 2025 in two private fertility centers: Instituto Bernabéu (Alicante) and Clínica Fertty (Barcelona).

Participants were recruited through convenience sampling among women who met the inclusion criteria established by the clinics for enrollment in the donation program. Participation was voluntary and anonymous, and all participants signed an informed consent form. The study was approved by the internal research committee of Instituto Bernabéu and registered under protocol code MR48 (March 15, 2024), in accordance with the ethical principles of the Declaration of Helsinki.

### Inclusion criteria

Women aged 18 to 33 years, with a body mass index (BMI) between 18 and 28, and without severe medical or psychiatric history.

### Instrument

The EMODON questionnaire (developed ad hoc for this study) (Supplementary Material 1) was designed by the research team in collaboration with mental health professionals and fertility specialists. The instrument assessed multiple dimensions of the donation experience, including motivations, concerns, perceived professional support, and emotional state during and after the process. It included closed-ended dichotomous and multiple-choice questions, as well as open-ended items to capture subjective opinions.

### Analysis

Descriptive statistics were applied to quantitative variables (frequencies, percentages, means, and standard deviations). Open-ended responses were analyzed using thematic qualitative analysis, identifying recurring patterns and emerging categories.

## RESULTS

### Sociodemographic profile

The sample consisted of 101 donors, with a mean age of 25 years (SD=3.88). A total of 62% had no children, 71% were Spanish nationals, and 58.3% had already completed two or more donation cycles (Table 1).

**Table 1:** Sociodemographic profile of oocyte donors (N=101)

Variable	Value
Mean age (years, SD)	25.0 (3.9)
Country of origin Spain Colombia Argentina Venezuela Uruguay Brazil Cuba Ecuador Paraguay Romania Russia Switzerland	71.3% 8.9% 4.0% 4.0% 3.0% 2.0% 2.0% 1.0% 1.0% 1.0% 1.0% 1.0%
Has children Yes No	37.6% 62.4%
Previous donation cycles, mean (SD)	2.9 (1.8)

### Motivations for donation

Altruistic motivation was reported by 95% of donors, while 61% indicated financial reasons. In addition, 20% expressed interest in obtaining information about their own fertility (Table 2).

**Table 2:** Motivations for donation

Motivation	Yes (%)	No (%)
Desire to help other women	95.0	5.0
Financial compensation	61.4	38.6
To learn about own fertility	19.8	80.2
Other reasons	11.9	88.1

### Concerns about donation

**Figure 1:**
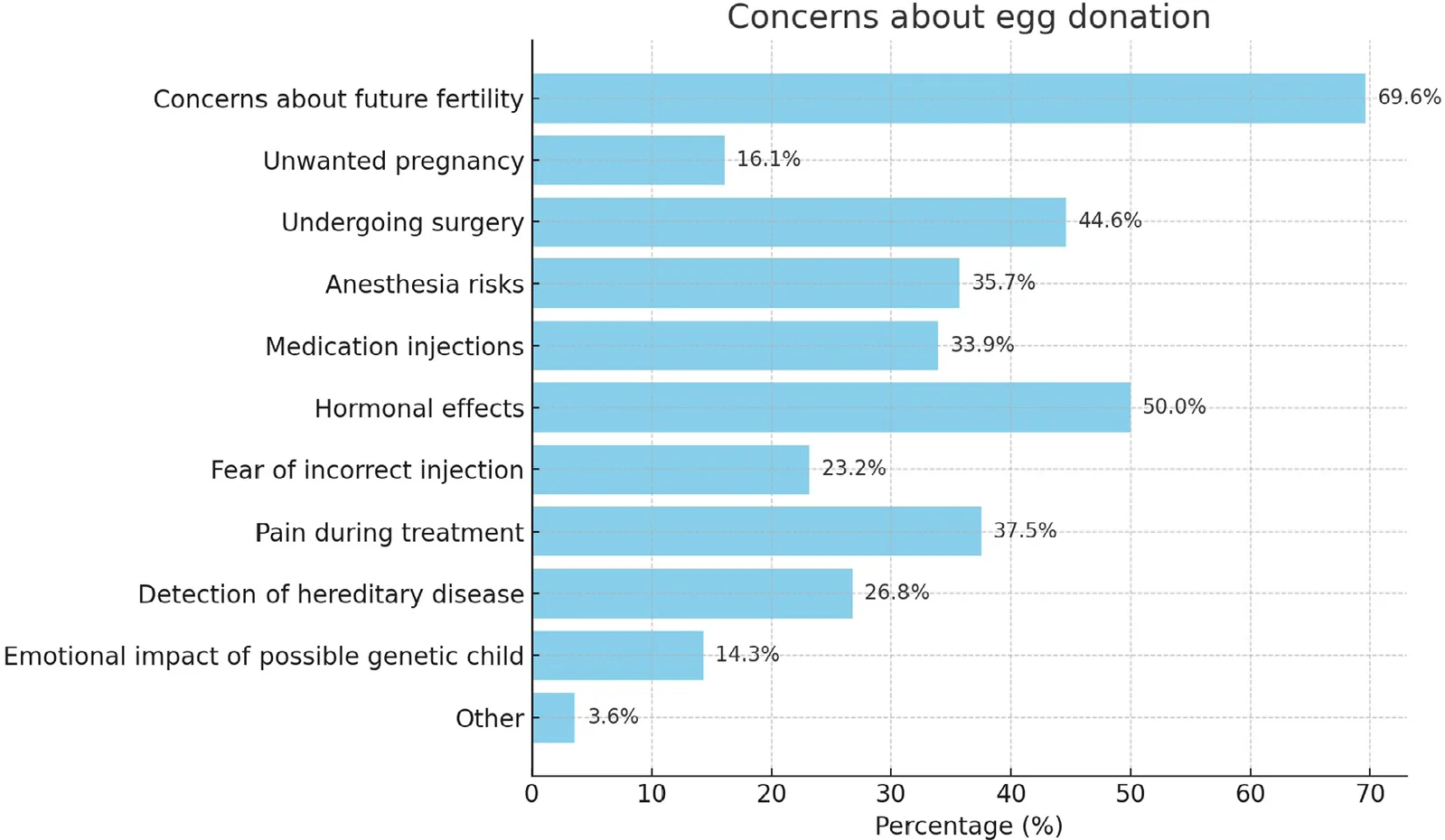
Concerns about egg donation

**Figure 2:**
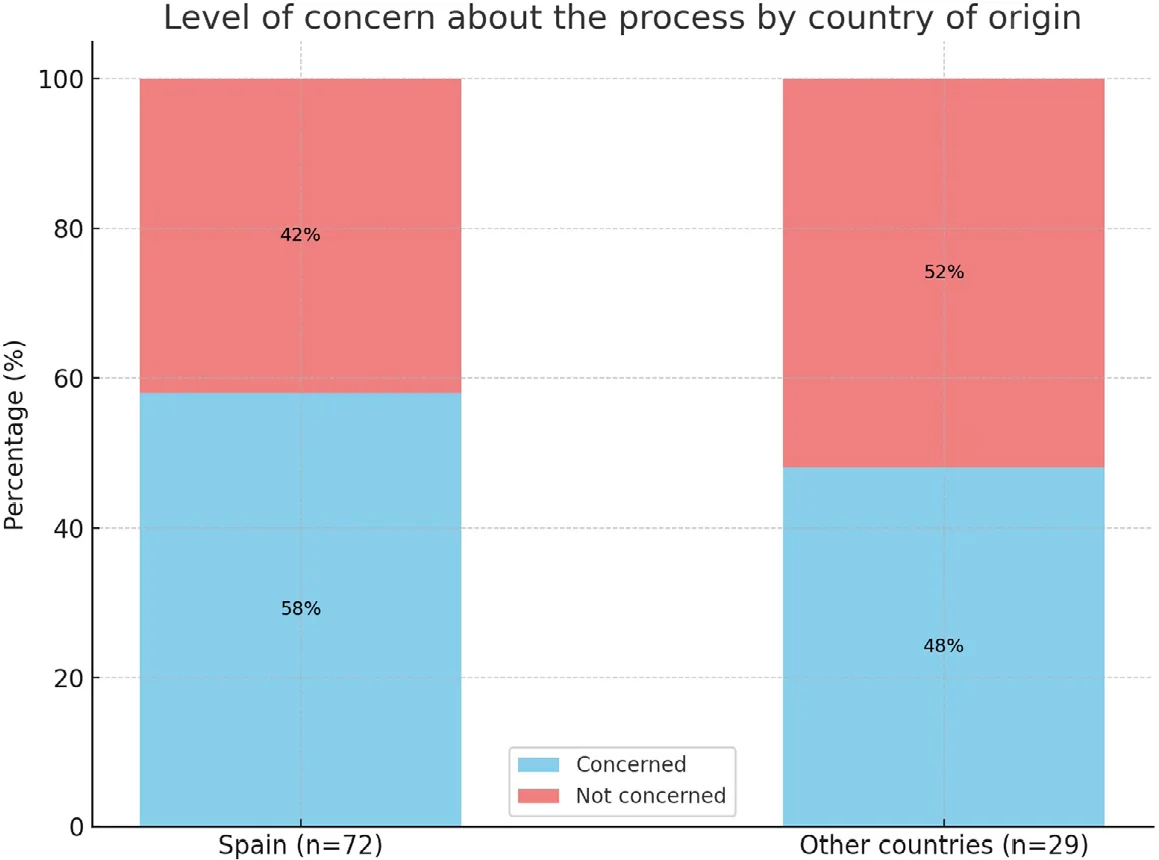
Level of concern about the process, by nationality

A total of 55% of donors reported doubts or fears prior to donation, mainly about future fertility (70%), surgical risks (45%), and hormonal effects (50%) (Figure 1). No statistically significant differences were found by nationality, although Spanish participants reported slightly higher levels of concern than foreign donors (58% *vs*. 48%) (Figure 2).

### Emotional state during and after donation

**Figure 3:**
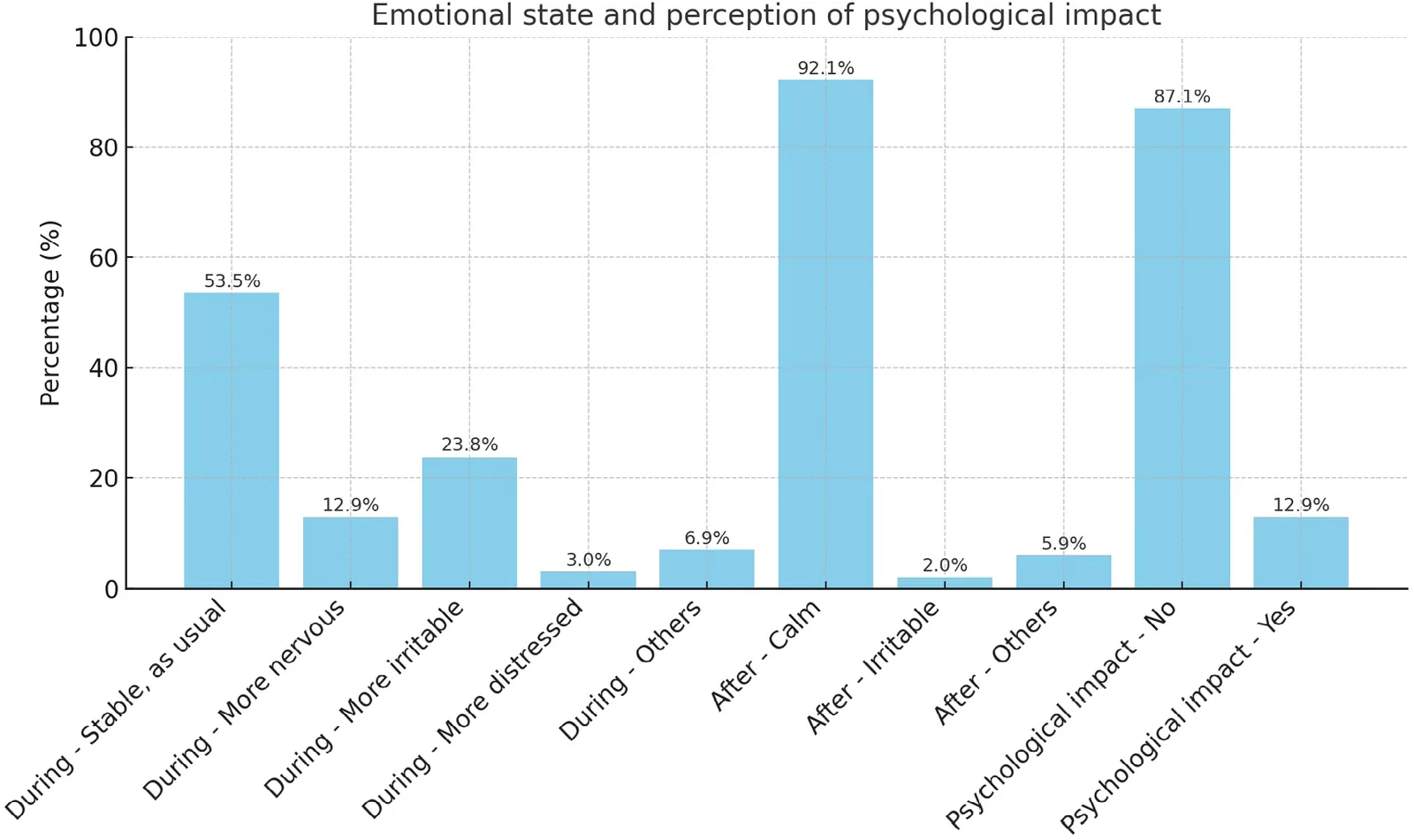
Emotional state during and after donation

**Figure 4:**
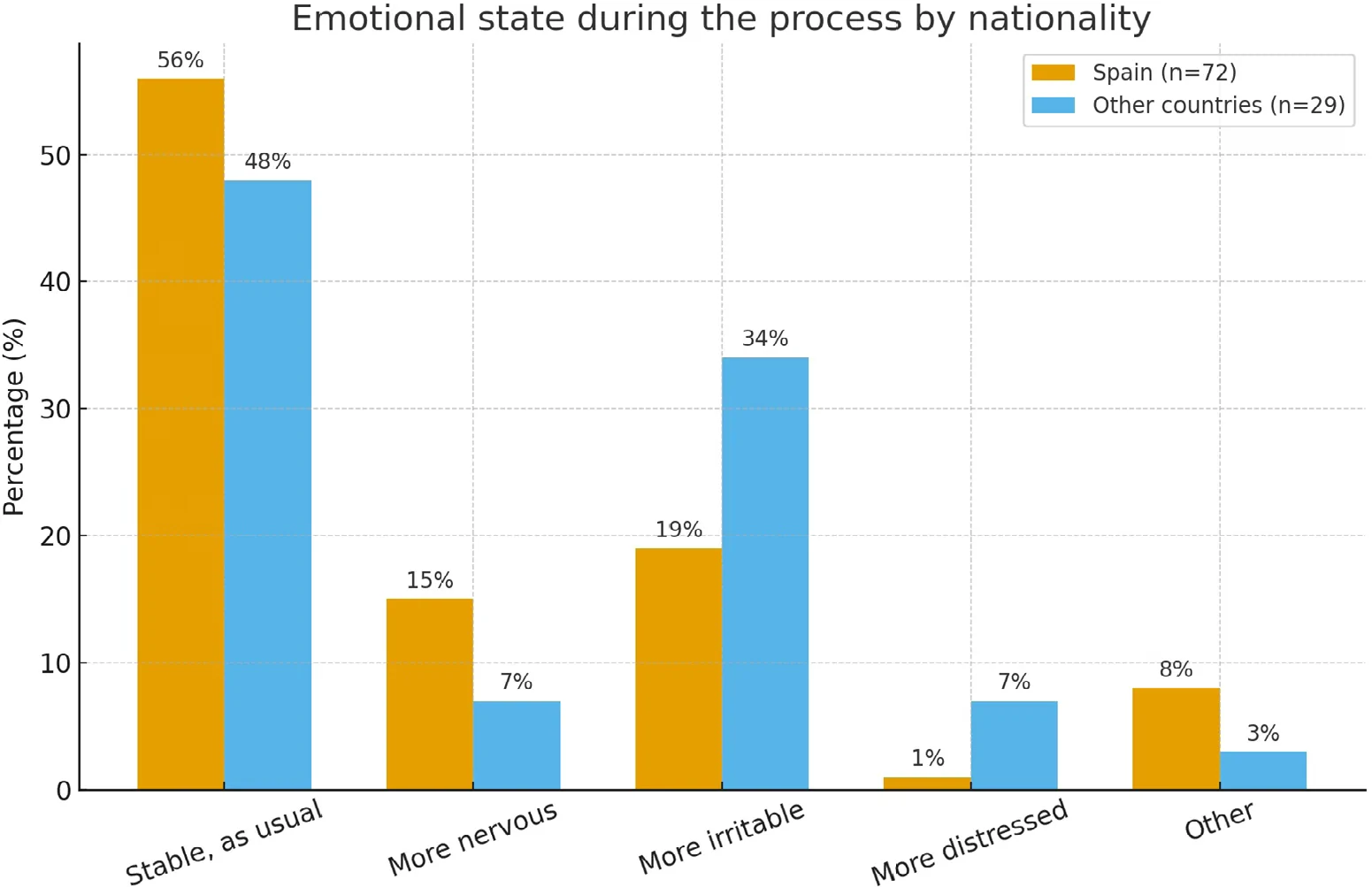
Emotional state during the process, by nationality

During stimulation, 54% felt emotionally stable, 24% reported increased irritability, and 13% felt more nervous. After donation, 92% reported feeling calm and satisfied (Figure 3). Notably, during the donation process, foreign donors reported higher levels of irritability compared to Spanish donors (34% *vs*. 19%) (Figure 4).

### Perceived emotional consequences

A minority (13%) reported experiencing negative emotional consequences such as sadness or persistent doubts.

### Professional support received

Almost all donors (99%) rated the clinical support positively (Table 3).

**Table 3:** Support received and perceived emotional effects

Variable	n	%
**Do you think the process had any psychological/emotional effect?** Yes No	13 88	12.9 87.1
**Did you feel supported by professionals?** Yes No	100 1	99.0 1.0

### Opinions on anonymity and age

**Figure 5:**
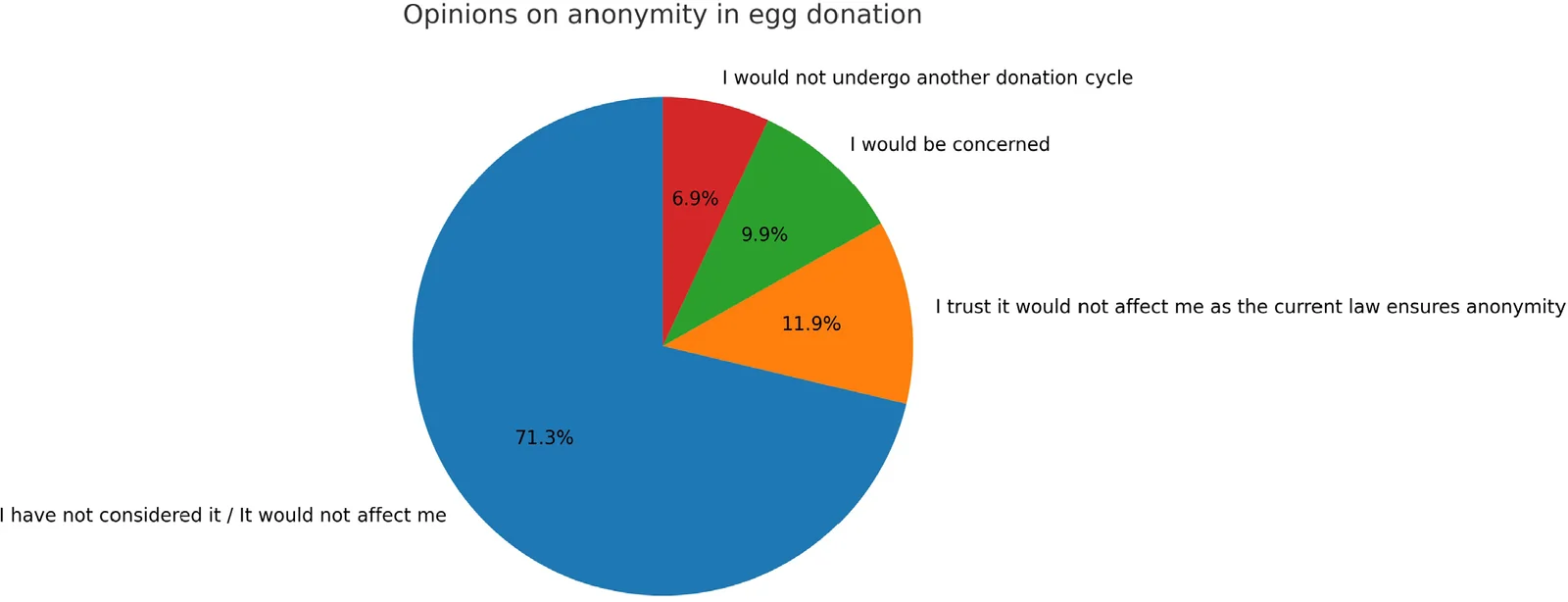
Opinions on anonymity in egg donation

A total of 71.3% reported that the elimination of anonymity would not have influenced their decision or that they had never considered it. However, 9.9% expressed concern, and 6.9% stated that they would not have donated again (Figure 5).

### Qualitative analysis of open-ended responses

Analysis of open responses revealed new categories that complemented the quantitative findings, offering a broader view of donors’ experiences and perceived needs.

1. **High satisfaction with emotional support:** 29 donors valued positively the care received from clinical staff, highlighting kindness, empathy, and trust.
2. **Need for better pre-treatment information:** 7 participants suggested providing updated information, including statistics, testimonials, and educational materials.
3. **Proposal for complementary psychological support:** up to 5 donors recommended optional free access to psychological services during and after treatment.
4. **Importance of humanized and personalized care:** 4 donors emphasized adapting the process to individual circumstances, such as flexible scheduling and mental load considerations.
5. **Desire for information about donation outcomes:** 3 donors expressed interest in knowing whether the donation was successful or if a child had been born healthy.

## DISCUSSION

Our findings were consistent with previous research highlighting the coexistence of altruistic and financial motivations among egg donors. Prior studies have emphasized that many women are driven by the desire to help others become mothers, although financial incentives remain a fundamental component of the process (Rivera, 2014; 2021a; 2021b; Lima *et al*., 2020; Hudson *et al*., 2019). Regarding anonymity, several works have revealed a similar trend: many donors do not fully consider the long-term implications, although some express unease at the possibility of being contacted in the future (Grau Rubio & Fernández Hawrylak, 2015; Pérez, 2019).

In contrast, other studies have highlighted shortcomings in the emotional support available to donors. Research from Argentina has criticized that medical monitoring often does not include adequate psychological care and that, unlike recipients, donors usually receive no follow-up support (Ariza, 2016). Similarly, Chardón (2015) noted that clinical approaches to gamete donation tend to focus on biomedical aspects, leaving aside the subjective dimension of donors. These findings contrast with our results, where most participants positively evaluated the professional support they received, emphasizing the closeness, empathy, and respect demonstrated throughout the process.

This study presented several strengths. It addressed an underexplored area: the emotional and psychological experiences of oocyte donors during the clinical process itself. It also integrated a mixed-methods approach (quantitative and qualitative), which allowed for a more comprehensive understanding of the donation experience.

Nevertheless, some limitations should be acknowledged. The sample was drawn from only two fertility centers, limiting the generalizability of the results to the wider donor population in Spain. In addition, most participants were Spanish nationals, which restricted the ability to make robust comparisons between women of different cultural backgrounds. Another limitation was that the questionnaire was administered at the end of the treatment, a moment when donors’ emotional perceptions may have been influenced by the immediate experience of ovarian puncture and its outcomes. This timing could have affected the sincerity or intensity of some responses, generating a potential bias.

## CONCLUSION

This study confirmed that oocyte donation was, overall, an emotionally well-tolerated experience, driven largely by altruistic motivations but also influenced by financial considerations. While most participants did not report emotional distress, a small subgroup expressed sadness or doubts, highlighting the need for targeted psychological support in certain cases. The high level of satisfaction with professional care reinforced the importance of an empathetic clinical environment. Furthermore, the growing social acceptance of oocyte donation was evident, although some stigmas remained. Finally, the issue of anonymity continued to generate diverse views, underlining the need for further exploration of its ethical and emotional implications from the donors’ perspective.

Future research should include longitudinal studies to examine how donors’ psychological states evolved over time, paying attention to persistent emotions such as the sense of having an unknown child or concerns about long-term fertility. It would also be worthwhile to explore whether certain sociodemographic variables-such as age, country of origin, educational level, or family situation-influenced the emotional experience of donation. Expanding the sample to include more centers across Spain would have allowed for a more diverse and representative population. Finally, future studies could reformulate some key questionnaire items, such as altruistic motivation, by posing more concrete questions (e.g., “Would you donate if no financial compensation were provided?”) in order to elicit more candid responses.

## Figures and Tables

**Supplementary Material 1:** EMODON Questionnaire (Last Revision: June 2024)

EMODON STUDIO

**Title of the study**	Study on motivations and emotional experiences in egg donors in Spain EMODON Study: Emotional Donors
**Protocol code**	MR48
**Promoter**	Instituto Bernabéu / Fertty Clinic
**Principal Investigators**	Marta Guerri Pons and Natalia Romera Agulló

**Age**		
**Country of origin**		
**Do you have any children?**	YES	NO
**Have you donated eggs more than once? If so, how many times?**		

**What motivated you to donate eggs? (You can select more than one option.)**	The possibility of helping other women	
	Financial compensation	
	Knowing if I am fertile	
	Others	

**How long did it take from the time you decided to donate until you completed the process?**	Between one and two months	
	Between two and four months	
	More than four months	

**Did you have any doubts when making the decision?**	YES	NO
**Have you shared your intention to donate with anyone (family, friends, etc.)?**	YES	NO
**If the previous answer is affirmative, have you felt supported?**	YES	NO
**Before starting the egg donation process, did you have any concerns or fears?**	YES	NO

**If the previous answer is yes, please indicate what your main concerns were (multiple options may be selected)**	
Possible changes in my fertility in the future.	
The possibility of an unwanted pregnancy during treatment.	
The fact of going through an operating room.	
The risks or side effects of anesthesia.	
The medication injections.	
The effect of hormones on my body.	
Not injecting myself properly	
Possible pain during treatment.	
The possibility of being diagnosed with a serious hereditary disease.	

The emotional impact of knowing that there could be a child genetically related to me in the world.	
Others (please specify). _________________________________________________________________________	

**How would you describe your emotional state during the donation process? (multiple options can be selected)**	Stable, as always	
	More nervous	
	More irritable	
	More distressed	
	Others	
**And after the donation process?** **(multiple options can be selected)**	Peaceful	
	Nervous	
	Irritable	
	Distressed	
	Others	

**Do you think the donation process has had any impact** **on your emotional or psychological well-being?**	Yes
	No
**Did you feel supported by the professionals?**	Yes
	No

	**I'**ve never thought about it.

**If the law were to change and anonymity were to disappear, how would you feel? (multiple options can be selected)**	I trust that it would not affect me because the current law is anonymous.	
	It wouldn't influence me.	
	I would be worried	
	I would not perform a new donation cycle	

**What changes would you suggest to improve emotional and psychological support for egg donors?**
**Any other comments or suggestions you'**d like to share?
